# Effect of Modulating Activity of DLPFC and Gender on Search Behavior: A tDCS Experiment

**DOI:** 10.3389/fnhum.2018.00325

**Published:** 2018-08-21

**Authors:** Xiaolan Yang, Yiyang Lin, Mei Gao, Xuejun Jin

**Affiliations:** ^1^School of Business and Management, Shanghai International Studies University, Shanghai, China; ^2^Academy of Financial Research, Zhejiang University, Hangzhou, China; ^3^College of Economics, Zhejiang University, Hangzhou, China

**Keywords:** search behavior, dorsolateral prefrontal cortex, transcranial direct current stimulation, risk attitude, gender difference

## Abstract

Studies of search behavior have shown that individuals stop searching earlier and accept a lower point than predicted by the optimal, risk-neutral stopping rule. This behavior may be related to individual risk preferences. Studies have also found correlativity between risk preferences and the dorsolateral prefrontal cortex (DLPFC). As risk attitude plays a crucial role in search behavior, we studied whether modulating the activity of DLPFC, by using a transcranial direct current stimulation (tDCS) device, can change individual search behavior. We performed a sequential search task in which subjects decided when to accept a point randomly drawn from a uniform distribution. A total of 49 subjects (23 females, mean age = 21.84 ± 2.09 years, all right-handed) were recruited at Zhejiang University from May 2017 to September 2017. They repeated the task in 80 trials and received the stimulation at the end of the 40th trial. The results showed that after receiving right anodal/left cathodal stimulation, subjects increased their searching duration, which led to an increase in their accepted point from 778.17 to 826.12. That is, the subjects may have changed their risk attitude to search for a higher acceptable point and received a higher benefit. In addition, the effect of stimulation on search behavior was mainly driven by the female subjects rather than by the male subjects: the female subjects significantly increased their accepted point from 764.15 to 809.17 after right anodal/left cathodal stimulation, while the male subjects increased their accepted point from 794.18 to 845.49, but the change was not significant.

## Introduction

Search behavior is evident in many areas, such as job searches (Cox and Oaxaca, 1989, 2000), shopping choices and investment decisions. The literature suggests that search behavior plays a crucial role in international trade (Besedes, 2008), mutual fund flows (Sirri and Tufano, 1998) and house prices (Ihlanfeldt and Mayock, 2012).

Studies have examined search behavior under different experimental designs. For example, Holt (2005) designed a search game where the point distribution was known and the search cost was constant. In another study, Cox and Oaxaca (2008) analyzed changes in optimal behavior along with changes in interest rates, subsidy, risk, cost, probability and horizon in the experiment. Viefers (2012) added uncertainty to the point distribution.

Many studies on search behavior have focused on search duration and the reservation point. For search duration, experimental studies have shown that individuals stop searching earlier than the duration predicted by the optimal and risk-neutral assumption (Schunk and Winter, 2009). The average search duration is also shorter when there is ambiguity about the point distribution than when the point distribution is known (Asano et al., 2011). However, subjects normally change their own reservation point under different conditions. For example, the reservation point tends to be lower when the true point distribution is unknown to subjects than when the point distribution is clear (Asano et al., 2015). Especially in the labor market, subjects lower their reservation wages if they have to wait an uncertain amount of time for offers to arrive (Brown et al., 2011).

Risk attitude is one of the major factors affecting search behavior. Holt (2005) studied search behavior based on the assumption of risk neutrality. Cox and Oaxaca (2008) found that the assumption of risk aversion better explained search behavior. Evidence has shown that heterogeneity in search behavior is linked to heterogeneity in individual preferences (Schunk and Winter, 2009). For example, ambiguity can notably affect the search behavior of risk-averse subjects but not of risk-neutral or risk-prone subjects (Asano et al., 2011). Additionally, research has observed gender differences in risk attitude, with women tending to be more risk averse than men in both gain and loss frames (Croson and Gneezy, 2009).

To predict search behavior with precision, different search models have been constructed, such as the real options model (Maart et al., 2011) and the reference point updating model (Schunk and Winter, 2009). However, the search duration suggested by the real options model is shorter than its actual duration (Maart et al., 2011), and the reference point updating model is still unable to explain how people form and update reference points in dynamic choice situations (Schunk and Winter, 2009). Because of individual heterogeneity, no model can explain individual search behavior and perfectly predict actual search decisions. As a result, the decision-making process in search behavior remains uncertain. Specifically, studies have found correlativity between search behavior and risk preference (Holt, 2005; Cox and Oaxaca, 2008; Schunk and Winter, 2009; Asano et al., 2011), while the causal relationship remains unclear.

Noninvasive brain stimulation (NIBS) techniques have been widely used for studying the physiology of the central nervous system and identifying the functional role of specific brain structures (Dayan et al., 2013). These techniques can reveal the causal relationship between brain activity and individual behavior. Transcranial direct current stimulation (tDCS) and transcranial magnetic stimulation (TMS) are the two most commonly used forms of NIBS. Both of them can identify causal links between specific brain structures supporting cognitive, affective, sensory and motor functions (Dayan et al., 2013). Therefore, it is necessary to conduct further research on brain activity during search behavior by using a tDCS or TMS device, which can reveal the causal relationship between search behavior and individual heterogeneities, including the heterogeneity of risk preferences and gender differences.

Search behavior involves many decision-making processes, which are determined by the activity of the cerebral cortex, especially the prefrontal cortex. Neuroimaging studies have shown evidence of a relationship between the decision-making process and the dorsolateral prefrontal cortex (DLPFC). For example, Fleck et al. (2006) used a functional magnetic resonance imaging (fMRI) device and found that right DLPFC activity was greater for low-confidence than for high-confidence decisions in episodic retrieval and visual perception tasks. More recently, brain stimulation techniques have been increasingly used to investigate how modulating the activity of the DLPFC may affect individual decision-making processes and risk attitudes. Some researchers have found that both right anodal/left cathodal and left anodal/right cathodal tDCS over the DLPFC can reduce the participants’ degree of risk aversion (Ye et al., 2015b) and increase the propensity for risk-taking among marijuana users (Boggio et al., 2010). However, Fecteau et al. (2007a) indicated that participants receiving bilateral DLPFC tDCS adopted a risk-averse response style during ambiguous decision making. Ye et al. (2015a) found that the participants tended to be risk seeking in the gain frame and risk averse in the loss frame after the right anodal/left cathodal tDCS over the DLPFC. For the TMS study, Knoch et al. (2006) found that subjects were more risk-taking after receiving 1 Hz rTMS over the right DLPFC when facing a complex risk task involving calculation of the level of risk and balance of benefit and risk. Subjects performing self-control behaviors in making intertemporal choices became more impatient after receiving 1 Hz rTMS over the left DLPFC (Figner et al., 2010). In short, NIBS can induce more cautious or riskier behaviors (Levasseur-Moreau and Fecteau, 2012). Because modulating the activity of the DLPFC by tDCS or TMS could change subjects’ risk preference, and risk preference plays an important role in search behavior, it is meaningful to investigate whether DLPFC could affect risky decision-making behaviors under uncertainty in the search game. This could allow us to better understand individual search behavior from a neuroscience perspective.

Gender differences have been widely discussed in the tDCS research on different prefrontal cortexes. For example, after anodal tDCS over the medial prefrontal cortex (mPFC), the ability to explain and predict other people’s mental states is enhanced in female subjects but not in males (Adenzato et al., 2017). Additionally, after receiving anodal tDCS over the ventral prefrontal cortex (VPC), female subjects tended to significantly increase utilitarian responses in tasks involving moral judgment, while males showed no significant difference (Fumagalli et al., 2010). Research on DLPFC has found that females improve their accuracy in verbal working memory (WM) after active right DLPFC anodal stimulation in the highest WM load condition, while males benefit more from left DLPFC stimulation (Meiron and Lavidor, 2013). Therefore, this study takes into account gender-related differences.

In this study, we investigated the casual relationship between DLPFC activity and search behavior. We performed a sequential search task. In the task, subjects decided when to accept the point at which a distribution was certain. Once subjects accepted the point, that trial was concluded, and the accepted point was converted into a payment; otherwise, subjects had to pay a constant cost for waiting for the next new point. Each trial continued indefinitely until a given point was accepted. We adopted a pre–post design and compared the subjects’ average search duration, average accepted point and average search income before and after different stimulation treatments. We aimed to test whether any types of stimulation could change subjects’ search behavior and to find causal relationships between DLPFC activity and search behavior. Gender differences were considered in our study.

## Materials and Methods

### Subjects

A total of 52 subjects (27 males, mean age = 22.30 ± 2.21; 25 females, mean age = 21.44 ± 1.88 years; 50 right-handed) were recruited at Zhejiang University from different majors via an advertisement posted on the school’s bulletin board system. The subjects were excluded if: (i) they did not understand the procedure; or (ii) they were left-handed or were left-handed before correction but now are right-handed. Based on these criteria, three subjects were excluded and 49 subjects (26 males, mean age = 22.31 ± 2.30; 23 females, mean age = 21.30 ± 1.72 years; all right-handed) remained. The subjects were randomly assigned to receive right anodal/left cathodal tDCS (*n* = 15, eight females), left anodal/right cathodal tDCS (*n* = 17, seven females), or sham stimulation (*n* = 17, eight females). The experiment lasted approximately 70 min and the average payment to the subjects was 49.19 CNY (approximately 7.81 USD)^1^. This study was carried out in accordance with the recommendations of the Zhejiang University ethics committee. The protocol was approved by the Zhejiang University ethics committee. All subjects gave written informed consent in accordance with the Declaration of Helsinki. None of the subjects reported any adverse side effects regarding pain on the scalp or headaches after the experiment.

### Transcranial Direct Current Stimulation (tDCS)

tDCS is a NIBS technique delivered by a battery-driven stimulator (multichannel noninvasive wireless tDCS neurostimulator, Starlab, Spain). A pair of saline-soaked sponge electrodes (5 cm × 7 cm) were fixed on the scalp of the participant using a rubber belt. We then applied a constant 2 mA current flow lasting for 20 min with 30 s of ramp up and down via the electrodes (Boggio et al., 2008; Fregni et al., 2008; Nitsche et al., 2008; Vanderhasselt et al., 2013). There were no physiological injuries to any of the participants. The tDCS technique facilitates neural excitability depending on electrode polarity. The anodal electrode enhances cortical excitability while the cathodal electrode weakens it (Nitsche and Paulus, 2000). As in a previous study (Gandiga et al., 2006), the current delivered in the sham stimulation only lasted for 30 s once it reached 2 mA. This constant but perceptible stimulation makes the subjects equate it with a regular process of stimulation.

**Figure 1:**
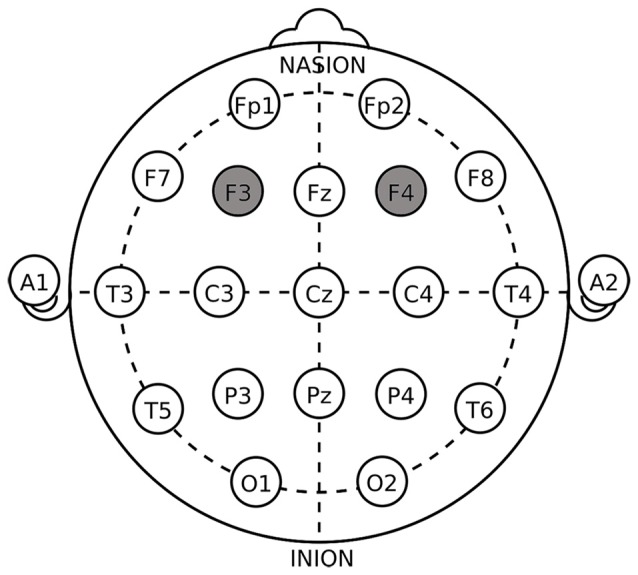
Electrode placements in dorsolateral prefrontal cortex (DLPFC) stimulations.

Electrodes placed over F3 and F4 affect the DLPFC area (Fecteau et al., 2007a,b; Boggio et al., 2009). Figure 1 shows that the anodal (cathodal) electrode was placed over the right F4 and the cathodal (anodal) electrode was placed over the left F3, following the right anodal/left cathodal (left anodal/right cathodal) treatment based on the International 10-20 System for electrode placement.

### Experimental Design

#### The Search Game

**Figure 2:**
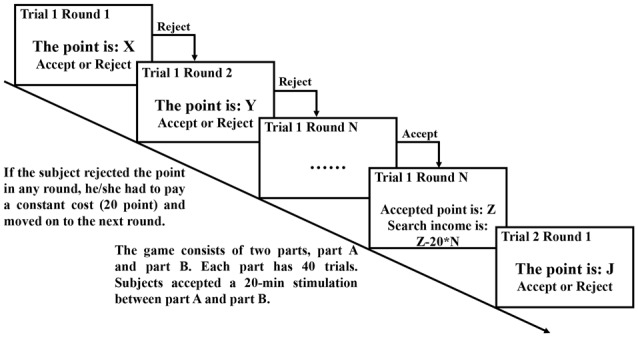
Schematic representation of the experimental design. In part A, we used the computer to generate 40 realizations for 40 trials from the uniform distribution (0, 1,000) before the game and presented the same realization to all of the subjects in the game. In part B, the same realizations of points as part A were used in 30 trials in a different order and 10 new realizations of points from the same uniform distribution (0, 1,000) were generated before the game and used in the remaining 10 trials.

The search game (Figure 2) was based on the experimental design of Holt (2005) and Asano et al. (2015). The game consisted of two parts, part A and part B. The subjects made decisions in each part. In each part, the subject faced 40 trials including unlimited rounds. In the first round of each trial, a point was drawn randomly from a uniform distribution with a lower bound of 0 and an upper bound of 1,000 by a computer^2^. The subject was expected to choose to click either the “accept” or “reject” button after observing the given point on the screen. Once the subject accepted the point, the trial was concluded and the accepted point was converted into a payment. Otherwise, he or she had to pay a constant cost and moved on to the next round in which a point was again drawn from the same point distribution. The subject continued to search in this manner until a given point was accepted. Recall was not allowed. In the game, 100 experimental points could be converted to 1.50 CNY and the constant cost was 20 points. For example, in one trial, a subject rejected the given points in the first three rounds and accepted the point of 852 in the fourth round. The search duration was four rounds, the total search cost was 80 points, and the search income (payment) was 11.58 CNY. The decision task in part B was similar to part A^3^. Part B used the same realizations of points as part A in 30 trials in a different order and used 10 new realizations in the remaining 10 trials.

The subjects then received a 20-min stimulation before part B. When the 40 trials in part B were concluded, one trial in part A and another trial in part B were randomly selected. Then, we calculated the payoffs of the two trials to determine the total payment for each subject.

#### Procedure

The experiment was conducted with the software z-Tree (Fischbacher, 2007). At the beginning of the experiment, each subject was provided with instructions. The subjects were informed that: (i) they would not incur any losses from the search task; (ii) they would earn an attendance fee of 20.00 CNY (3.18 USD); and (iii) other payoffs were determined by their decisions made in the experiment. After a public reading of the instructions, three pilot trials were conducted to facilitate subjects to practice the task. Then, part A, including 40 trials, was started. The trials were conducted one at a time. After the 40 trials in part A were concluded, the experimenters placed tDCS devices on the subjects’ heads for the 20-min stimulation. The subjects were reminded to sit comfortably and relax. When the stimulation ended, the devices were removed. New experimental instructions were provided in another public reading, and part B of the experiment commenced, again with z-Tree software. When the 40 trials in part B were concluded, one trial in part A and another trial in part B were randomly selected by the computer to determine the subjects’ payoffs. The final payment was a combination of the show-up fee and the payoffs from the two parts. Finally, each subject completed a questionnaire before finally receiving their payment. This questionnaire contained 15 questions regarding personal information such as gender, age, major, place of origin, household income, consumption expenditures and the experimental process. The questionnaire information is summarized in Supplementary Table S1.

#### Data Analysis

We aimed to test whether the subjects’ search behavior would be changed after the stimulation between part A and part B. We randomly selected 30 trials out of total 40 trials in part A and repeated them in different orders in part B. Data analysis was only focused on these selected 30 trials both in part A and part B. To investigate the subjects’ search behavior, we measured the subjects’ average search duration, average accepted point, and average search income, and compared them before and after the right anodal/left cathodal stimulation, the left anodal/right cathodal stimulation, and the sham stimulation. Statistical analyses were performed using SPSS statistical software (version 20).

Repeated-measures analysis of variance (ANOVA) and a paired *t*-test were used in statistical analysis. Repeated-measures ANOVA with parameters (search duration, accepted point, and search income) and time (before and after stimulation) were used as with-subject factors, while stimulation types (right anodal/left cathodal, left anodal/right cathodal and sham) served as between-subject factors and were used to test the influence of stimulation. The sample was divided into male and female groups and a paired *t*-test was used to examine differences in single variables (search duration, accepted point and search income) before and after stimulations in each group. By comparing the effect of stimulation in the two groups, we could test the influence of gender differences on search behavior. Supplementary Table S1 shows all the experimental data.

## Results

### The Optimal Reservation Point in Search Behavior

In our experiment, each point was drawn randomly from a uniform distribution (0, 1,000) and the search cost was 20 points. Following Holt (2005), we used an expected value to find the optimal reservation point on the assumption of risk neutrality. The optimal reservation point can be found by locating the point at which the expected benefit of another search is equal to the search cost (Holt, 2005).

Suppose the current draw is 800 in our experiment and we consider the expected gains from searching. There is a 4/5 chance that the next draw is 800 or below, in which case the net gain is 0 and the expected value of the gain is (4/5)0 = 0. There is a 1/5 chance that the next draw is more than 800 and the net gain on average is half of the distance from 800 to 1,000, i.e., 100. Then, the expected value of the gain is (1/5)*(100) = 20 points. Therefore, the total expected benefit of another search is 20 points. Any lower current draw produces an expected benefit from a further search that is above 20 points, and any higher current draw produces a lower expected benefit. Obviously, 800 is the optimal reservation point with the assumption of risk neutrality in which the expected benefit of another search equals the search cost. For a risk-averse person, the optimal reservation point is lower than 800 because he or she prefers to stop at 800, which represents a sure thing and does not involve the uncertainty of searching for a new point. For a risk-seeking person, the optimal reservation point is higher than 800. Finally, for any risk-neutral subject, the optimal search strategy is simply to keep searching until a draw of more than 800 appears.

The means of the average accepted point before and after stimulation in the experiment are summarized in Table 1. The means of the average accepted point before the three types of stimulation were lower than predicted by the optimal, risk-neutral stopping rule, which is consistent with Schunk and Winter (2009). In addition, the mean of the average accepted point after the right anodal/left cathodal stimulation was higher than the means after the other two types of stimulation. This result indicates that the subjects who received the right anodal/left cathodal stimulation may have been more risk seeking and tended to accept a higher point than other subjects. This is consistent with Ye et al. (2015a), who found that subjects tended to be risk seeking in the gain frame after the right anodal/left cathodal tDCS over the DLPFC.

**Table 1:** The means of average accepted point before and after three types of stimulation.

	Treatment	Mean (point)	Distance (point)
	Sham	792.80	−7.20
Before stimulation	L+/R−	788.57	−11.43
	R+/L−	778.17	−21.83
	Sham	814.79	14.79
After stimulation	L+/R−	812.15	12.15
	R+/L−	826.12	26.12

*L+/R−, Left anodal/Right cathodal; R+/L−, Right anodal/Left cathodal; Distance equals the mean of average accepted point minus the optimal reservation point 800. Average accepted points before and after stimulation were calculated based on the experimental data, where there were 30 trials before and after stimulation*.

### Effect of tDCS on Search Behavior

There was no significant difference in the subjects’ average search duration, average accepted point, or average search income in different treatments before the stimulation (one-way ANOVA; search duration: *F*_(2,46)_ = 0.023, *p* = 0.977; accepted point: *F*_(2,46)_ = 0.104, *p* = 0.902; search income: *F*_(2,46)_ = 0.162, *p* = 0.851). This demonstrated that the subjects’ search behavior was not different across the treatments before stimulations.

**Figure 3:**
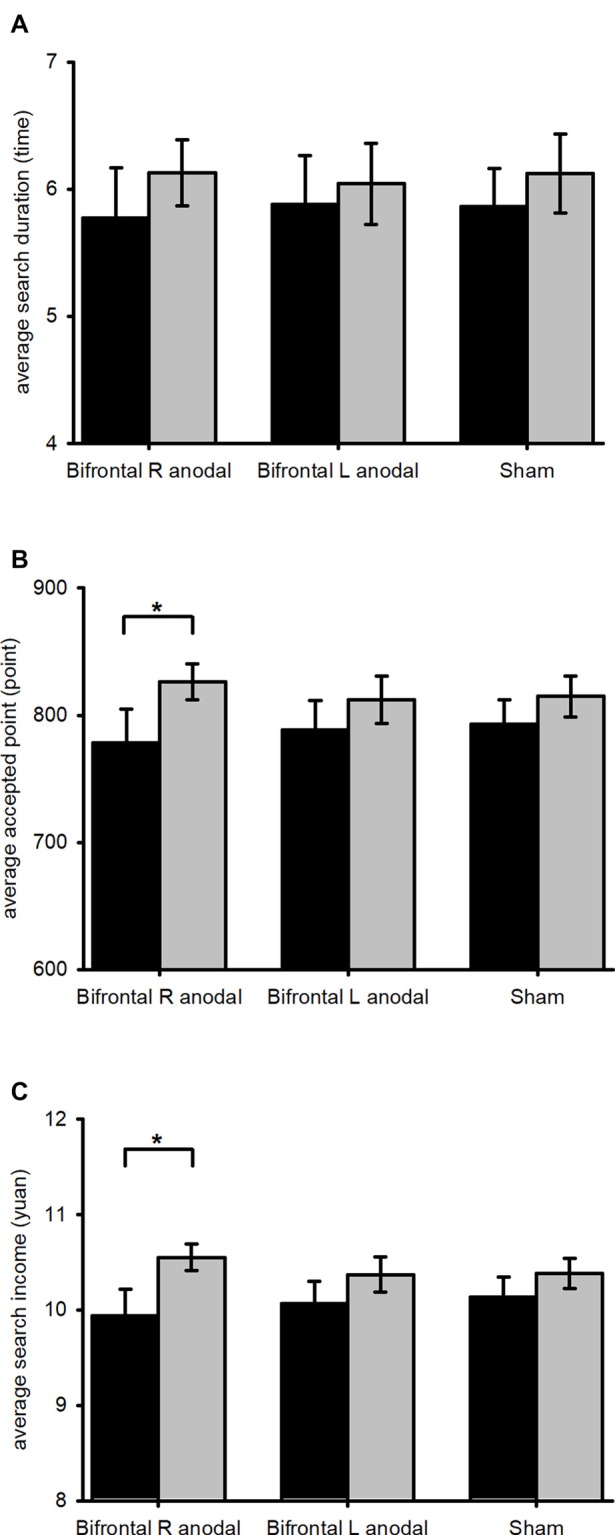
The average values of search duration, accepted income and search income before and after stimulation. Black bars, pre-transcranial direct current stimulation (pre-tDCS); gray bars, post-tDCS. Error bars indicate 95% confidence intervals. Subjects’ average search duration showed no significant difference after any types of stimulation **(A)**. Subjects’ average accepted point and average search income were significantly higher after right anodal/left cathodal tDCS **(B,C)**. *P < 0.05.

To test whether the stimulation of tDCS changed the subjects’ search behavior in different treatments, we applied repeated-measures ANOVA with parameters (average search duration, average accepted point, and average search income) and time (before and after stimulation) as with-subject factors, while stimulation types served as between-subject factors. We found a significant effect of the interaction between time and parameter (*F*_(1,46)_ = 14.180, *p* < 0.001). Simple main effect tests showed diverse effects of different treatments on different parameters (Figure 3). The subjects’ average search duration showed no significant difference before and after the stimulation (sham: *F*_(1,46)_ = 1.200, *p* = 0.279; left anodal/right cathodal: *F*_(1,46)_ = 0.450, *p* = 0.506; right anodal/left cathodal: *F*_(1,46)_ = 1.945, *p* = 0.170). The subjects’ average accepted point and average search income were significantly higher after the right anodal/left cathodal stimulation (accepted point: *F*_(1,46)_ = 7.795, *p* = 0.008; search income: *F*_(1,46)_ = 10.598, *p* = 0.002), but there was no significant difference after the left anodal/right cathodal stimulation (accepted point: *F*_(1,46)_ = 2.137, *p* = 0.151; search income: *F*_(1,46)_ = 2.983, *p* = 0.091) or the sham stimulation (accepted point: *F*_(1,46)_ = 1.858, *p* = 0.179; search income: *F*_(1,46)_ = 2.020, *p* = 0.162). These results indicated that subjects tended to increase the accepted point and obtained a higher search income after receiving right anodal/left cathodal stimulation.

To make our conclusion more robust, we used the experimental data and calculated the medians of search duration, accepted point and search income before and after the stimulation. Then, we compared them by repeated-measures ANOVA again. A significant effect of the interaction between time and parameter was again found (*F*_(1,46)_ = 10.018, *p* = 0.003). Similar effects of different treatments on the accepted point and search income were observed. After receiving the right anodal/left cathodal treatment, the subjects’ median of accepted point and median of search income were significantly higher (accepted point: *F*_(1,46)_ = 7.435, *p* = 0.009; search income: *F*_(1,46)_ = 9.566, *p* = 0.003), but there was no significant difference in the subjects’ accepted point and search income before and after the left anodal/right cathodal stimulation (accepted point: *F*_(1,46)_ = 1.211, *p* = 0.277; search income: *F*_(1,46)_ = 1.375, *p* = 0.247) or the sham stimulation (accepted point: *F*_(1,46)_ = 1.221, *p* = 0.275; search income: *F*_(1,46)_ = 1.303, *p* = 0.260). The results of medians were consistent with the results of the means, which strongly verified our conclusion.

In conclusion, we found that the accepted point was significantly higher after the right anodal/left cathodal stimulation. More specifically, the subjects’ accepted point was slightly lower than the optimal reservation point before the stimulation but exceeded the optimal reservation point after the right anodal/left cathodal stimulation. This significant difference in the accepted point before and after the stimulation may be related to a change in risk attitude. After receiving the right anodal/left cathodal tDCS over the DLPFC, subjects tended to be risk seeking (Ye et al., 2015a), and increased their reservation point in the game. As the accepted point significantly increased after the stimulation, search income also increased significantly.

### Gender Differences

Finally, we tested whether the gender of subjects affected search behavior. One-way ANOVAs showed no significant effect of gender on search duration (*F*_(1,47)_ = 1.603, *p* = 0.212), accepted point (*F*_(1,47)_ = 2.433, *p* = 0.126), or search income (*F*_(1,47)_ = 2.759, *p* = 0.103) before the stimulation, but found a significant effect of gender on accepted point (*F*_(1,47)_ = 2.975, *p* = 0.091) and search income (*F*_(1,47)_ = 3.657, *p* = 0.062) after the stimulation at the level of 10% significance. Additionally, we added gender to the repeated-measures ANOVA and a main effect of gender (*F*_(1,43)_ = 3.349, *p* = 0.074) was observed.

**Figure 4:**
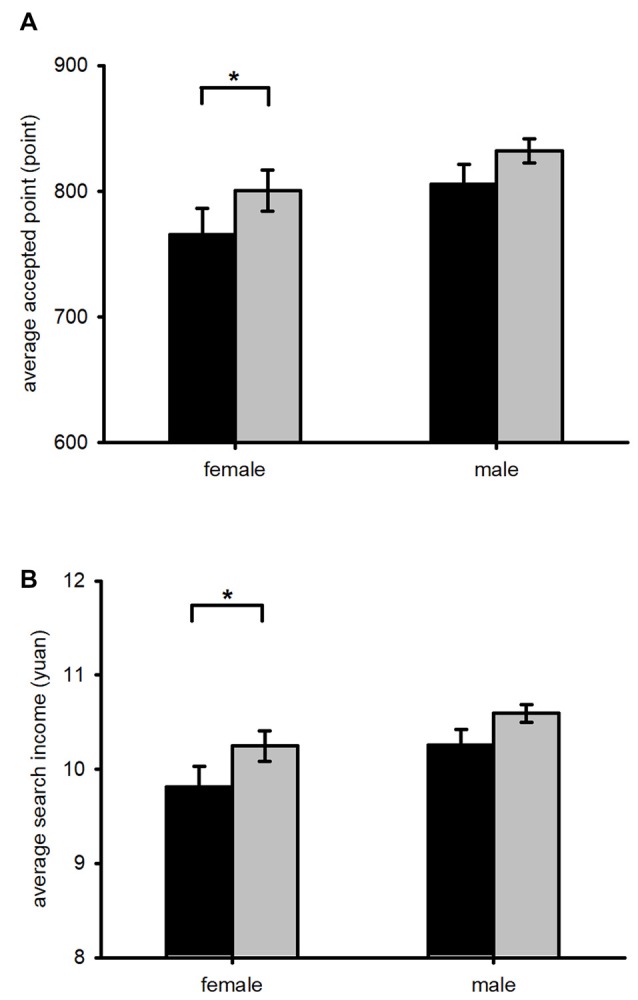
The gender difference before and after receiving right anodal/left cathodal tDCS over DLPFC. Black bars, pre-tDCS; gray bars, post-tDCS. Error bars indicate 95% confidence intervals. Female subjects significantly increased accepted points and search income after the right anodal/left cathodal stimulation, while male subjects showed no significant difference after the same stimulation. *P < 0.05.

To further test the relationship between gender differences and stimulation types in search behavior, we divided the entire sample into two groups: males (*n* = 26) and females (*n* = 23). We applied a paired *t*-test to distinguish the differences between the two groups (Figure 4). There was no significant difference in the male subjects’ search behavior before or after the three types of stimulation. There was no significant difference in the female subjects’ search behavior after the left anodal/right cathodal stimulation or the sham stimulation. After receiving right anodal/left cathodal stimulation, the female subjects significantly increased their average accepted point (*t*_(1,7)_ = −2.793, *p* = 0.027) and average search income (*t*_(1,7)_ = −3.443, *p* = 0.011), but the average search duration showed no significant difference (*t*_(1,7)_ = −0.878, *p* = 0.409). These results demonstrated that the female subjects tended to significantly increase their accepted point and gain higher search income after the right anodal/left cathodal tDCS, while the male subjects showed no significant difference. This suggests that the significant differences of accepted point and search income in the total sample before and after the right anodal/left cathodal stimulation were mainly attributable to the female subjects.

In addition, a gender difference in risk preference was observed before the stimulation. The mean of the average accepted point in the female group was 765.60, which was lower than the optimal reservation point, while the mean of the average accepted point in the male group was 805.65, which was higher than predicted by the optimal rule. This difference suggests that even before the stimulation the female subjects were risk averse and the male subjects were risk seeking, consistent with Croson and Gneezy (2009) and Ye et al. (2015a). In our experiment, we found that gender differences were also significant after the right anodal/left cathodal stimulation. The female subjects were more sensitive to the stimulation and became risk seeking after the right anodal/left cathodal stimulation, while the male subjects showed no significant difference in risk attitude after the same stimulation. As a result, after the right anodal/left cathodal stimulation, the female subjects significantly increased their accepted point and gained higher search income, while the male subjects showed no significant difference.

## Discussion

In this study, we tested the effect of modulating the activity of DLPFC on search behavior. By comparing the average values of search duration, accepted point, and search income before and after the stimulation, we found that using the right anodal/left cathodal tDCS over DLPFC significantly increased the subjects’ accepted point and search income. Furthermore, this study demonstrated that there was a gender difference after the stimulation. The accepted point and search income were significantly higher after the right anodal/left cathodal stimulation in the female group, while there was no significant difference in the male group. This revealed that the effect of tDCS on search behavior was mainly driven by the female subjects rather than by the male subjects.

Previous tDCS research has studied individual decision-making behavior involving risk attitudes. We further constructed a sequential search task with uncertainty and incorporated tDCS devices into the task to analyze subjects’ search behavior. We aimed to reveal the causal relationship between DLPFC activity and search behavior and find the exact effect of different types of stimulation on the subjects’ behavior. The between-subjects design normally lacks statistical power because there is heterogeneity among different subjects. Hence, we adopted a within-subject design to avoid heterogeneity among subjects and compared search behavior before and after the stimulation. However, the learning effect may be significant. Thus, we added three pilot trials to facilitate subjects to become familiarized with the task. This can stabilize the baseline performance thus reducing learning effects. We also randomized the order of the trials. Our results showed that there was a significant difference after the right anodal/left cathodal stimulation but not after the left anodal/right cathodal stimulation or the sham stimulation. Therefore, the learning effect might have been reduced in our search game.

In our experiment, the subjects significantly increased their accepted point and obtained higher search income after receiving right anodal/left cathodal stimulation. Considerable literature has shown that people normally stop searching earlier than predicted by the optimal, risk-neutral stopping rule (Schunk and Winter, 2009). The risk aversion assumption is more consistent with individual search behavior than the risk neutral assumption (Cox and Oaxaca, 2008). In fact, risk attitude does play an important role in search behavior (Cox and Oaxaca, 1989; Asano et al., 2011). Previous tDCS studies have shown that subjects tend to choose more risky options after right anodal/left cathodal tDCS in the gain frame (Ye et al., 2015a). Subjects’ degrees of risk aversion may be reduced after both right anodal/left cathodal and left anodal/right cathodal tDCS over DLPFC (Ye et al., 2015b). Combined with the results from the literature, one possible explanation for our results is that subjects receiving right anodal/left cathodal stimulation were more risk seeking and had a higher reservation point, so they chose to stop searching late and significantly increased their accepted point, which was larger than the optimal reservation point. As a result, they also gained a significantly higher search income.

Studies in experimental economics have indicated that there are substantial gender differences regarding risk aversion (Croson and Gneezy, 2009; Ye et al., 2015a). These studies are consistent with our results indicating that males tended to be more risk seeking than females before the stimulation. Several possible reasons can explain the gender difference in risk taking. First, previous research shows that both men and women are overconfident, but men are more overconfident in their success in uncertain situations than women (Deaux and Farris, 1977; Lichtenstein et al., 1982; Lundeberg et al., 1994). Generally, men are more confident to gain a higher point in the next search and more risk seeking to spend more time searching for an acceptable point. Second, men are more likely to regard a risky situation as a challenge that calls for participation, while women treat it as a threat that encourages avoidance (Arch, 1993). Thus, when facing uncertainty in the search game, men tended to search for a longer time than women.

Moreover, we found that there were significant gender differences in search behavior after the stimulation. The female subjects significantly increased their accepted point and gained higher search income after the right anodal/left cathodal tDCS, while the male subjects showed no significant difference. Recent studies have found that bilateral tDCS stimulation over DLPFC altered individuals’ risk attitude (Ye et al., 2015a,b; Zheng et al., 2016). We may infer from our results that the female subjects became more risk seeking after increasing right DLPFC activity and decreasing left DLPFC involvement in search behavior, while the male subjects showed no significant difference in risk attitude after the same stimulation. This supports the idea that there are different brain activation patterns elicited in response to cognitive tasks between males and females (Bell et al., 2006). These results also suggest that females were responsible for the changes in the accepted points and search incomes before and after the right anodal/left cathodal tDCS over DLPFC.

In addition, executive functions have been widely studied in neuroscience and they can effortfully guide behavior towards a goal (Banich, 2009). Some studies have found that the DLPFC is important for executive function (Wagner et al., 2001; Forbes et al., 2014). A number of researchers have studied gender differences in executive function. Several researchers have found that women outperform men on tests of verbal memory (Weiss et al., 2003) and information processing (Majeres, 1990). Wanless et al. (2013) and Gestsdottir et al. (2014) found that girls tended to exhibit more inhibitory control than boys during childhood. Search behavior reflects a role of executive functions, which are a set of mental skills that help you get things done. The present study provides new evidence for gender differences in executive functions. Our findings also support the view that DLPFC plays an important role in execution function.

The limitations in our study primarily concern the focality of tDCS. Since electrode montage could favor current spread across the stimulated cortices, it remains unclear whether the stimulation effects of tDCS were the result of selective modulation of the target area or the result of the inevitable widespread and nonselective modulation over the cortex (Sellaro et al., 2016). Unilateral stimulation is necessary to make our results more robust in future research.

## Data Availability

All datasets (generated/analyzed) for this study are included in the manuscript and the supplementary files.

## Author Contributions

XY, YL, MG and XJ designed the experiment, wrote the manuscript, revised the manuscript and finally approved the version to be published. YL and MG performed the experiment and drew the figures. XY and YL analyzed the data.

## Conflict of Interest Statement

The authors declare that the research was conducted in the absence of any commercial or financial relationships that could be construed as a potential conflict of interest.

## Footnotes

^1^We used the exchange rate of 6.2962 CNY to 1 USD on February 5, 2018.

^2^Before the experiment, we used the computer to generate 40 realizations for 40 trials from the uniform distribution (0, 1,000) and presented the same realization to all of the subjects in the experiment. Adopting the above method, sampling error was controlled and the subjects’ decisions were compared with one other.

^3^Among those trials in part B, the same realizations of points as part A were used in 30 trials in a different order. This controlled for sampling error and, hence, facilitated the comparison between the subjects’ decisions in the two parts. No subject was informed about the relationship between the two parts. Ten new realizations of points from the same uniform distribution (0, 1000) were used in the remaining 10 trials to prevent subjects from discovering the relationship between the two parts. After the experiment, the 30 trials used both in part A and part B were selected as the required experimental data.

## Supplementary Material

TABLE S1 Experimental data and questionnaire information. The means and medians of search duration, accepted point and search income before and after the stimulation are listed in the table. The data from Q2 column to Q13 column correspond to the questions on the questionnaire.
