# Supplementary material for: Effect of Modulating Activity of DLPFC and Gender on Search Behavior: A tDCS Experiment
Source: Front Hum Neurosci. 2018 Aug 21;12:325. doi: 10.3389/fnhum.2018.00325 (PMC6110849; doi:10.3389/fnhum.2018.00325)
Supplement: Supplementary file 2 [file Presentation_1.pdf]

Questionnaire

Hello! Thank you very much for your great support and cooperation in this experiment!

In order to further study, we hope you fill out the following questionnaire truthfully. Your answer will be of great help to our research. The questionnaire is anonymous and the data obtained is for research only. There will not be any adverse effect on you. Thank you!

1. Personal basic information:

Experiment code: Gender: Age: Major: Grade:

Birthplace: Province/city County Township

2. Are you an only child? (Y/N)
3. Where is your domicile? (Country/City)
4. Your family's annual income?

A. Less than ￥50,000 B. ￥50,000-￥100,000 C. ￥100,000-￥200,000

D. ￥200,000-￥500,000 E. More than ￥500,000

5. Your monthly consumption expenditure?

A. Less than ￥1,000 B. ￥1,000-￥1,500 C. ￥1,500-￥2,000

D. ￥2,000-￥5,000 E. More than ￥5,000

6. Do you understand our experimental task? (Y/N)
7. Recall your previous decision-making process, please use A-E to indicate your degree of agreement with the following two questions.

① Every decision I made was calculated accurately.

A. Strongly disagree B. Mildly disagree C. Agree and disagree equally

D. Mildly agree E. Strongly agree

② Every decision I made was only a rough estimate and it depends on my feeling.

A. Strongly disagree B. Mildly disagree C. Agree and disagree equally

D. Mildly agree E. Strongly disagree

8. In experiment A, which point is the lowest one do you think you would like to accept?
9. In experiment B, which point is the lowest one do you think you would like to accept?
10. Have you ever participated in a similar experiment (an experiment in economics or psychology)? (Y/N)
11. Have you ever taken part in any kind of part-time job? (Y/N)
12. How many students, who participate in the experiment at the same time do you know?

A. None B. One student C. Two students

D. Three students E. More than three students

13. Your dominant hand is? (Left hand/Right hand) (If your dominant hand was left hand before correction but now is right hand, please also choose left hand)
14. How do you feel when you receive the stimulation?

15. What are your suggestions for our experiment?

Thank you again for your great support and assistance!
